# The complete mitochondrial genome of *Leptomantella tonkinae* (Hebard, 1920) (Mantodea: Leptomantellidae) and its phylogeny

**DOI:** 10.1080/23802359.2022.2080025

**Published:** 2022-06-16

**Authors:** Yi-Jie Lin, Yu-Yang Zhao, Yong-Mei Yang, Wan-Ting Jin, Ling-Na Cai, Kenneth B. Storey, Jia-Yong Zhang, Dan-Na Yu

**Affiliations:** a College of Chemistry and Life Science, Zhejiang Normal University, Jinhua, China; b Key Lab of Wildlife Biotechnology, Conservation and Utilization of Zhejiang Province, Zhejiang Normal University, Jinhua, China; c Department of Biology, Carleton University, Ottawa, Canada

**Keywords:** *Leptomantella tonkinae*, Leptomantellidae, mitogenome, phylogenetic relationship

## Abstract

The complete mitochondrial (mt) genome of *Leptomantella tonkinae* (Hebard, 1920) was 15,527 bp in length and contained 13 protein-coding genes, 22 transfer RNAs, two ribosomal RNAs, and one control region. The gene arrangement of mt genome of *L. tonkinae* was identical to the primitive mantis. The overall AT content of the mt genome was 74%. In ML and BI phylogenetic analyses, the monophyly of Leptomantellidae was robustly supported and the clade of Leptomantellidae is a sister clade to the group of (Gonypetidae+(Leptomantellidae+(Amorphoscelidae+Nanomantidae))).

Praying mantises are a major group of predatory insects, with over 2500 species/subspecies in 29 families and 60 subfamilies according to the website http://Mantodea.SpeciesFile.org (Otte et al. 2021). Leptomantellidae is originally included in Iridopterygidae as a genus (*Leptomantella*), which has recently been promoted to a new family that included four genera (Ehrmann 2002; Wieland 2013; Schwarz and Roy 2019). Leptomantellids can be distinguished from other mantises because of some special characteristics including juxta-ocular bulges distinct, rounded; wings mostly hyaline or subhyaline; and the presence of four discoidal spines (Schwarz and Roy 2019). However, only one complete mitochondrial (mt) genome of Leptomantellidae (*Leptomantella albella* GenBank: KJ463364) was available in NCBI (Wang, Yu, et al. 2016a), which hindered the phylogenetic relationship of Leptomantellidae. Hence, to enrich the molecular dataset of the Leptomantellidae, we sequenced the complete mt genome of *Leptomantella tonkinae* (Hebard, 1920) (Mantodea: Leptomantellidae). The mt genome was deposited in GenBank with accession no. OK480879. The BI and ML phylogenetic trees were constructed by using the 13 PCGs of mt genomes, which aimed to discuss the phylogenetic relationship of Leptomantellidae.

*Leptomantella tonkinae* is not a protected insect species, so no permission for scientific research in China is needed. Our sample (GXJX20170714-1) was collected from Jinxiu Yao Autonomous County, Guangxi Zhuang Autonomous Region, China (24.13°N, 110.18°E). Based on the taxonomic system of Otte, the sample was identified as *L. tonkinae* by its morphometric features and was stored at −40 °C in the Animal Specimen Museum, College of Chemistry and Life Sciences, Zhejiang Normal University, China. Whole genomic DNA was extracted from leg muscle tissue using an Ezup Column Animal Genomic DNA Purification Kit (Sangon Biotech Company, Shanghai, China) and stored at Zhang’s lab (http://mypage.zjnu.edu.cn/ZJY3/zh_CN/index.htm, Zhang JY, zhang3599533@163.com). The mt genome was amplified with several universal primers as described in Zhang et al. (2018a). To fill the gaps in previously obtained sequences, some species-specific primers were designed using Primer Premier 5.0 (Lalitha 2000). Finally, the DNA fragments were checked and assembled using DNASTAR Package v.7.1 (Burland 2000).

The complete mt genome of *L. tonkinae* was 15,527 bp in length and included 13 protein-coding genes (PCGs), 22 transfer RNAs (tRNAs), two ribosomal RNAs (rRNAs), and one control region (681 bp). The nucleotide composition of *L. tonkinae* was shown to be A: 37.9%, C: 15.7%, G: 10.3%, and T: 36.1%, with an overall AT% content of 74%. Furthermore, the AT-skew and GC-skew of the mt genome were 0.025 and −0.207, respectively, which was similar to the published mt genome of *L. albella* (Wang, Yu, et al. 2016a). Additionally, a 32 bp non-coding region located between *trnP* and *ND6* was also found in *L. albella* (Wang, Yu, et al. 2016a). Based on the mt genome, the genetic distance with the Kimura 2-pweremeters model between *L. albella* and *L. tonkinae* was calculated as 0.056 by MEGA7.0 (Kumar et al. 2016). This gene arrangement was identical to the primitive insect mt genomes (e.g. Ayivi et al. 2021; Guan et al. 2021; Xu et al. 2021; Zhang et al. 2021).

To discuss the phylogenetic position of *L. tonkinae*, one newly sequenced mantis mt genome as well as 68 previously sequenced mantis mt genomes were used in phylogenetic analyses (Cameron et al. 2006; Song et al. 2016; Wang, Yu, et al. 2016a; Wang, Hou, et al. 2016b; Ye et al. 2016; Tian et al. 2017; Zhang and Ye 2017; Zhang et al. 2018a, 2018b, 2018c, 2018d; Jia et al. 2019; Shi et al. 2019; Yan and Lin 2019; Zhang et al. 2019; Guan et al. 2020; Wang et al. 2020; Xu et al. 2021). In addition, two cockroach species, *Eupolyphaga sinensis* and *Cryptocercus kyebangensis* (Zhang et al. 2010) and two termites, *Termes hospes* (Dietrich and Brune 2016) and *Macrotermes barneyi* (Wei et al. 2012) were chosen as the outgroups. Based on the 13 PCGs, two types of phylogenetic trees were constructed using maximum-likelihood (ML) (Stamatakis 2014) and Bayesian inference (BI) (Huelsenbeck et al. 2001) methods via the MrBayes with a GTR + I+G model and RAxML 8.2.0 with a GTRGAMMAI model, respectively. In BI and ML trees (Figure 1), *Leptomantella tonkinae* was a sister clade to *L. albella*, and Leptomantellidae was a sister clade to the clade of (Amorphoscelidae + Nanomantidae). According to the results of Schwarz and Roy (2019), they supported the promotion of *Leptomantella* to the family level. This was consistent with previous studies that supported the monophyly of the families Nanomantidae, Toxoderidae, and Mantidae (Zhang et al. 2018a, 2018b, 2018c, 2018d, 2019; Xu et al. 2021). However, Hymenopodidae and Deroplatyidae were shown to be paraphyletic groups and the monophyly of the families Haaniidae and Deroplatyidae was not supported, which was also found in the results of Zhang et al. (2018a, 2018d). Hence, to explore these discrepancies, more mt genomes should be sequenced in the future.

**Figure 1.**
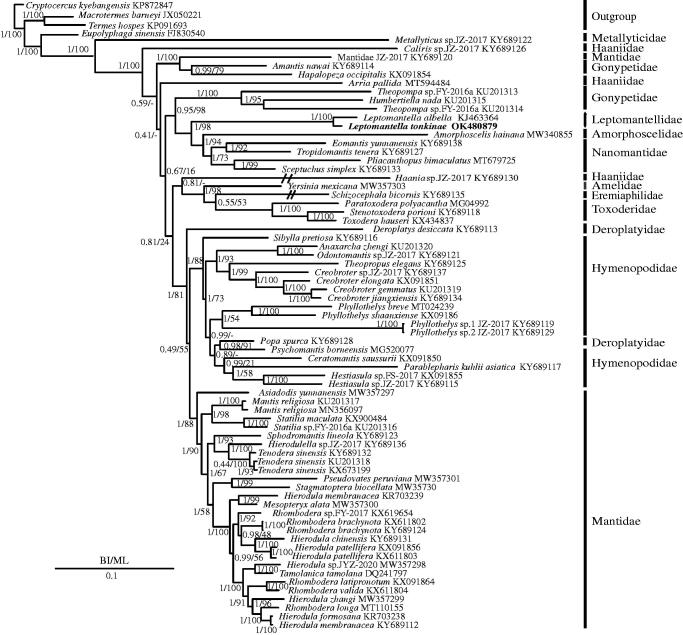
Phylogenetic tree of the relationships among 69 species of Mantodea inferred from BI analysis (A) and ML analysis (B) with two cockroaches (*Eupolyphaga sinensis*, *Cryptocercus kyebangensis*), and two termite (*Termes hospes*, *Macrotermes barneyi*) species chosen as outgroups. The GenBank accession numbers of all species are shown in the figure.

## Author contributions

All authors were involved in the conception and design, or analysis and interpretation of the data; Lin YJ, Zhao YY, Yang YM, Jin WT, and Cai LN were involved in the drafting of the paper; Storey KB, Zhang JY, and Yu DN were involved in revising it critically for intellectual content; and all authors were involved in the final approval of the version to be published; and that all authors agree to be accountable for all aspects of the work.

## Data Availability

The mitochondrial genome data that support the findings of this study are openly available in GenBank of NCBI at https://www.ncbi.nlm.nih.gov/nuccore/OK480879 under the accession no. OK480879. The mt genome was obtained by the Sanger method, so no associated ‘BioProject’, ‘SRA’, and ‘Bio-Sample’ numbers should be shown.
